# Dental age estimation: development and testing of a reference data set for Qatari children, adolescents, and young adults aged between 5 and 25 years

**DOI:** 10.1007/s12024-023-00587-5

**Published:** 2023-04-05

**Authors:** Noof Al-Obaidli, Najat Al-Hashimi, Victoria S. Lucas, Graham Roberts

**Affiliations:** 1 Department of Orthodontics, Guy’s Tower Wing, Great Maze Pond, Faculty of Dentistry, Oral & Craniofacial Sciences, King’s College London, Floor 25, Tower Wing, SE1 9RT London Bridge, London, UK; 2 Department of Orthodontics, Rumailah Hospital, Al Khaleej Street, 0097444397030 Doha, Qatar

**Keywords:** Reference data set, Tooth development stages, Dental age estimation, Qatari population, Demirjian tooth development stages

## Abstract

The purpose of this study is to establish and test a reference data set of dental development of Qatari subjects aged between 5 and 25 years. Radiographs of individuals aged between 5 and 25 years were re-used to establish a reference data set (RDS). A scheme comprising 8 tooth development stages (TDS) was used to assess all the teeth on the left side of the maxilla and mandible. The accuracy of dental age estimation (DAE) was tested with a separate sample of radiographs – the validation sample (VS) comprised 50 females and 50 males of known chronological age (CA). Dental panoramic tomographs (DPT) of 1,597 Qataris were assessed. The summary data for the individual TDS comprising the number (n-tds), mean ($\overline{\mathrm{x} }$-tds), standard deviation (sd-tds), 0th%-ile (the minimum), 25th%-ile, 50th%-ile (the median), 75th%-ile, and 100th%-ile (the maximum) were used to estimate the age of the VS subjects using the simple average method (SAM). There is a significant difference in dental age of 4.8 months in the female group when compared to the CA. The difference in the male group is 4.5 months. This shows similar differences to assessments of other ancestral or ethnic groups.

## Introduction

There has been a significant increase in illegal migration during the last 20 years. Many migrants do not have identification documents or a record of their date of birth. Age estimation (AE) is important for legal, social, forensic, and archaeological purposes.

Dental age estimation offers a biological state that mimics chronological age thus enabling the following:

1. Estimation of the chronological age (CA) of an individual with unknown date of birth or missing identification documents. In addition to the point estimate of age, it is important to indicate the uncertainty associated with the estimate. Age estimation of these individuals is necessary to establish eligibility for civil rights, education, or social benefits.
2. A further need for AE is in criminal proceedings where the judicial process is determined by the age of the accused.
3. To provide reference standards for different ethnic, ancestral, racial, and identifiable human groups.

The United Nations Convention on the Rights of the Child (UNCRC 1989) [1] is a widely recognised international treaty that has changed and improved the way children are perceived across the globe. Young people aged less than 18 years are cared for under regulations pertaining to minors. There are large numbers of refugees of uncertain age who do not have birth records. Age estimation is necessary to ensure that social care, healthcare, and education are appropriately provided. There is also a safeguarding issue because adult asylum seekers who are over 18 years but claiming to be under 18 years of age should not be domiciled in the same accommodation as minors. DAE may assist in cases of child trafficking. Underage girls have been forced into marriage, and in these cases, traffickers claim that the children are older than they appear.

Age estimation may be important in criminal proceedings as some criminals, to avoid being punished as adults, claim to be younger than they look. This is so these young adults serve a sentence under the juvenile penal code. In Qatar, the age of criminal responsibility for children is between 7 and 17 years of age. This is stipulated by the Supreme Judiciary Council in Qatar.

There have been many attempts to devise schemes to estimate the age of children [2, 3] In a paper published in 1973 [2] it was stated that “ … it should be remembered that the sample on which they are based is of entirely French-Canadian origin. The dental maturity scores for a given chronological age may well be greater or less in other populations …” This is an important and often overlooked statement.

The purpose of this study is to use extant dental panoramic tomographs (DPT) to prepare a reference data set (RDS) for Qatari children, adolescents, and young adults to create an ethnic-specific dataset to enable assessment of dental maturity in Qatari subjects and from this to estimate dental age (DA) with the associated range of uncertainty.

## Materials and methods

### Reference data set

The RDS comprised 1600 DPTs of female and male Qatari children, adolescents, and young adults. The DPTs were retrieved from the radiological archives of Rumailah Hospital and Al-Wakra Hospital outpatient departments as well as an orthodontic office. The chronological age of each subject was calculated using the date of birth and the date of radiographic exposure of the DPT. Digital copies of the DPTs were transferred from Qatar using a portable password protected storage device. This was to ensure and protect anonymity. Ethical approval was granted by the Hamad Medical Corporation Research Centre in Qatar [Research protocol #1518/15 “Dental Age Assessment” Ref No. MRC 1535/2016.].

#### Exclusion criteria

- Non-Qatari subjects
- Poor quality DPTs
- Subjects with a medical condition that may affect dental maturation

### Tooth development stages

**Fig. 1:**
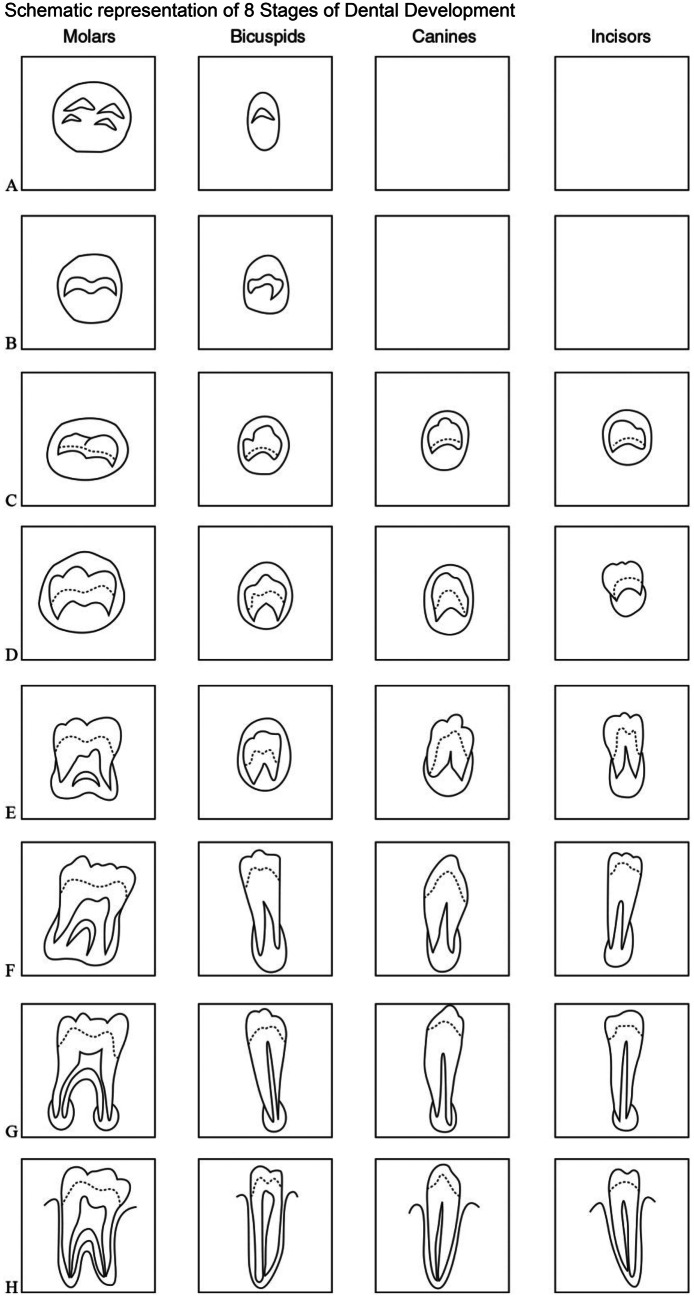
The 8 stages of dental development described by the Anglo-Canadian research team. These descriptions should be used when viewing dental panoramic tomographs to determine the stage of development from **A** to **H**

These were assessed using the 8 stage system described by Demirjian, Goldstein, and Tanner in 1973 (Fig. 1 and Table 1) [2]. The date of birth and sex was blinded from the assessor at the time of the assessment.

**Table 1:** Written guide of morphologically related descriptions of Demirjian’s TDS

**Tooth development stage (TDS)**	**Single rooted teeth and multi-rooted teeth [descriptions]**
**A**	In both uniradicular and multiradicular teeth, a beginning of calcification is seen at the superior level of the crypt in the form of an inverted cone or cones. There is no fusion of these calcified points
**B**	Fusion of the calcified points forms one or several cusps, which unite to give a regularly outlined occlusal surface
**C**	**a**. Enamel formation is complete at the occlusal surface. Its extension and convergence toward the cervical region is seen **b**. The beginning of a dentine deposit is seen **c**. The outline of the pulp shape has a curved shape at the occlusal border
**D**	**a**. Crown formation is complete down to the cemento-enamel junction **b**. The superior border of the pulp chamber in uniradicular teeth has a definite curved form, being concave towards the cervical region. The projection of the pulp horns, if present, gives an outline like an umbrella top. In molars, the pulp chamber has a trapezoid form **c**. Beginning of root formation is seen in the form of a radiopaque spicule
**E**	Uniradicular teeth **a**. The walls of the pulp chamber now form straight lines, whose continuity is broken by the presence of the pulp horn, which is larger than in the previous stage **b**. The root development is less than the crown Multiradicular teeth **a**. Initial formation of the radicular bifurcation is seen in the form of either a calcified point or a semilunar shape **b**. The root length is less than the crown height
**F**	Uniradicular teeth **a**. The walls of the pulp chamber now form a more or less isosceles triangle. The apex ends in a funnel shape **b**. Root development is equal to or greater than the crown Multiradicular Teeth **a**. The calcified region of the bifurcation has developed further down from its semilunar stage to give the roots a more definite and distinct outline, with funnel shaped endings **b**. The root length is equal to or greater than the crown height
**G**	**a**. The walls of the root canals are now parallel (distal root of molars) **b**. The apical ends of the root canals are still partially open
**H**	**a**. The apical end of the root canal is completely closed (distal root in molars) **b**. The periodontal membrane has a uniform width around the root and apex

**Fig. 2:**
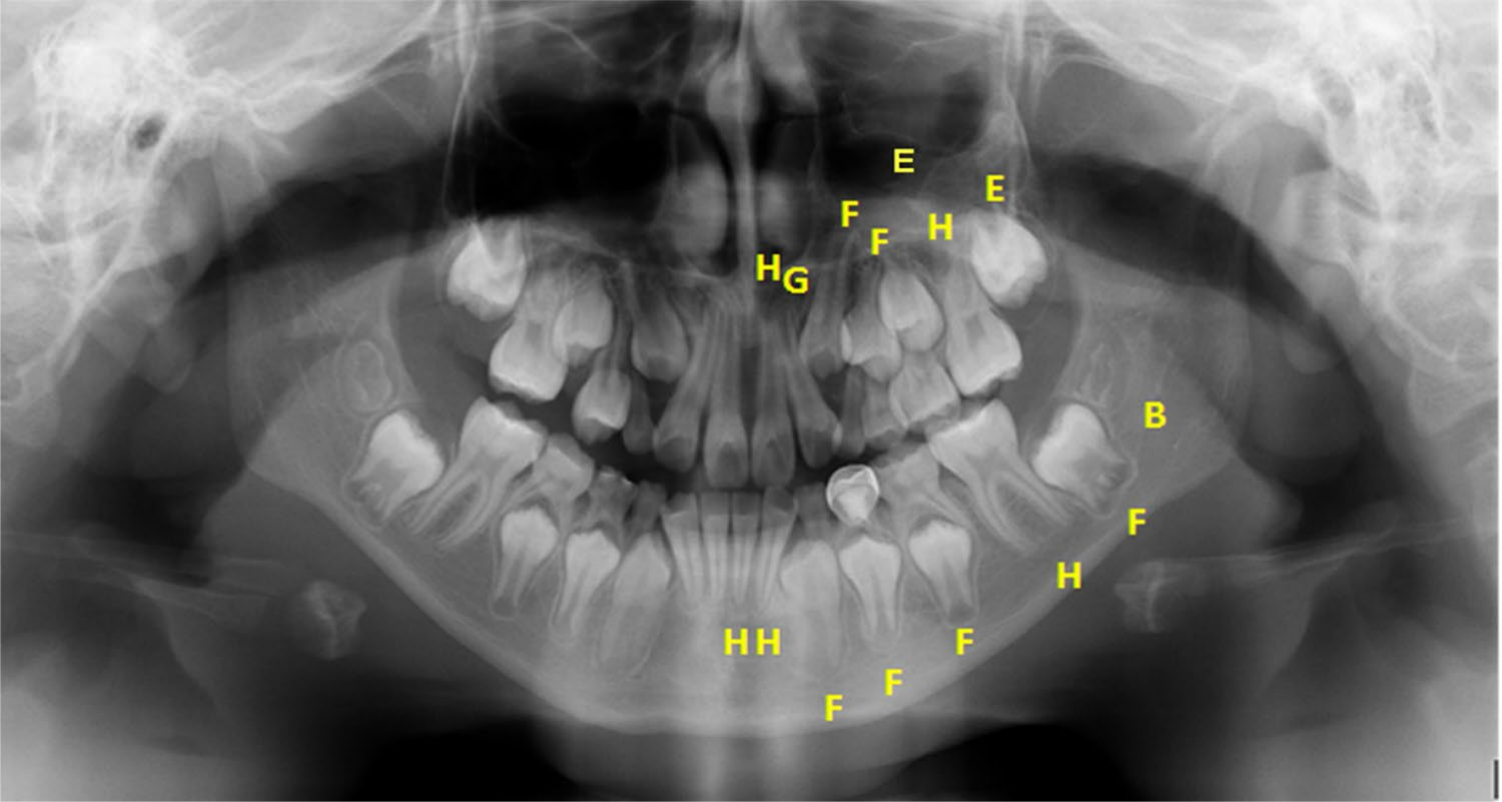
DPT for subject ID 47 female showing the TDS assessed for this subject. Teeth on the left side only are used. The tooth development stages are indicated in the upper case type on the image

The TDS were recorded on a specially designed form and then entered into a Microsoft Access database. The assessment included all the teeth on the left side of both the maxilla and mandible. Teeth that were fully developed (stage H) were recorded but *excluded* from calculations for DAE (Fig. 2).

### Observer agreement

The primary investigator (NAO) recorded the TDSs on the left side of 10 DPTs, comprising 160 assessments. These DPTs were separate from the main study. The assessments were on 2 occasions 2 weeks apart to determine within observer agreement (WOA). A second observer (VSL) assessed the same DPTs to determine between observer agreement (BOA). Both the WOA and the BOA were calculated using Cohen’s kappa [4], and the outcome index was assessed according to the categories suggested by Landis and Koch in 1977 [5].

### Data processing

The major part of the study was to establish a RDS for the young Qatari population. The TDSs (A through H) of each tooth present on a sample radiograph were entered into a Microsoft Access database. To extract data from the database, queries were created which included the following information: ID number, ethnicity, sex, and age at assessment (AaA) of each TDS.

These queries were exported to Excel and a small worksheet was created for each TDS for both females and males, for example, UL8Gf [Upper Left Third molar, Stage G, female]. Each of these queries comprised the dataset for that specific stage from which the number (n-tds), the mean ($\overline{\mathrm{x} }-\mathrm{tds}$), standard deviation (sd-tds), 0th%-ile (minimum), 25th%-ile, 50th%-ile (median), 75th%-ile, and the 100th%-ile (maximum) were calculated.

### Validation study

The second part of this work was to assess the accuracy of the method of DAE when utilizing the Qatari RDS. The validation sample (VS) comprised 100 DPTs of Qatari children and adolescents. There were 50 females and 50 males with known date of birth and date of radiographic exposure. These data were blinded from the assessor. The radiographs were separate from the RDS. The TDS from each tooth on the left side was entered into an Excel worksheet which semi-automatically looked up the n-tds, $\overline{\mathrm{x} }-\mathrm{tds}, \mathrm{and\;sd}-\mathrm{tds}$, from the RDS to calculate the DA to enable comparison of the CA with the DA for each VS subject [6]. When the 50 female and 50 male subjects had been assessed, CA and DA were compared using Student’s t test.

**Fig. 3:**
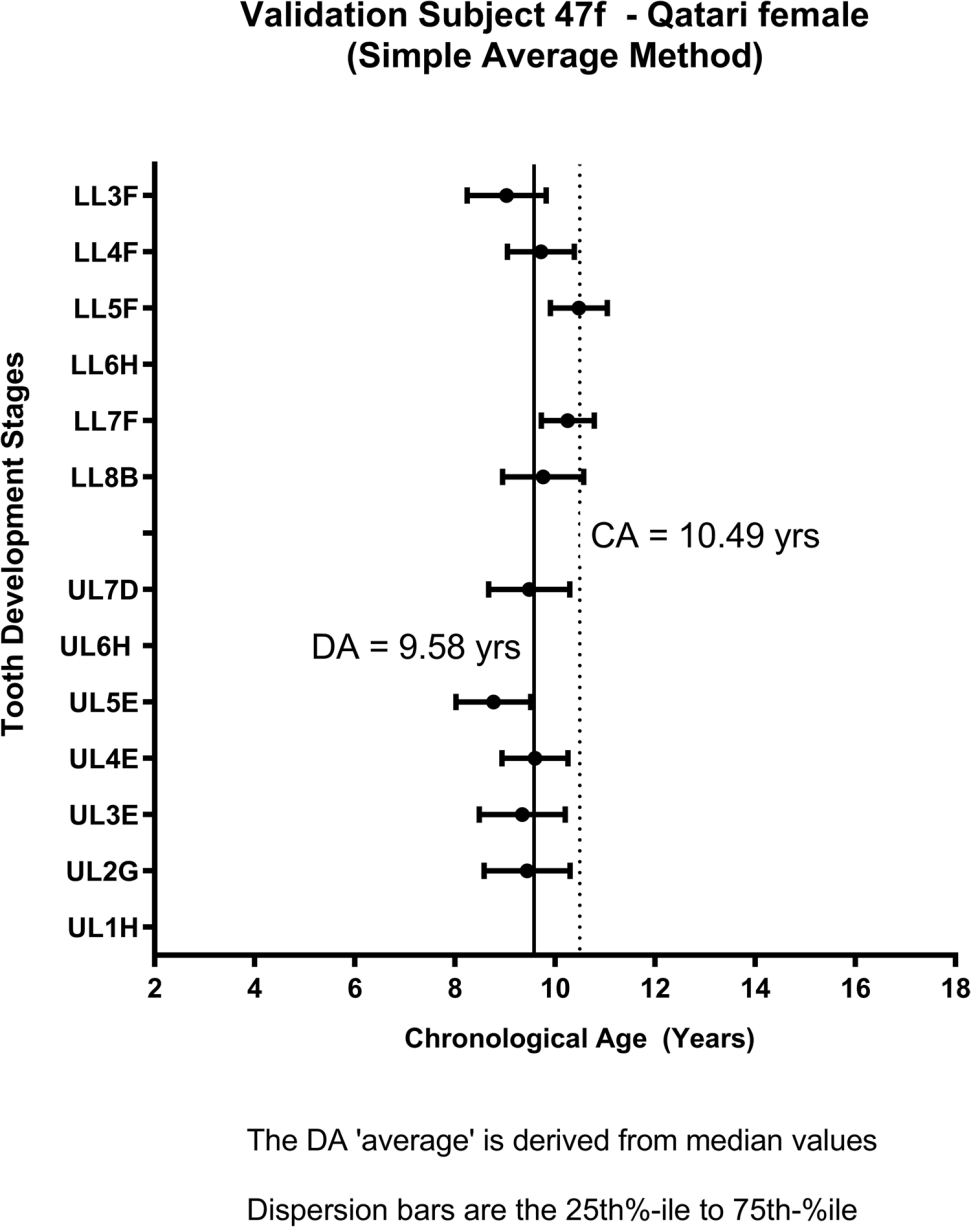
Forest plot showing the variation around the median value for DA using the 25th to 50th percentiles

**Fig. 4:**
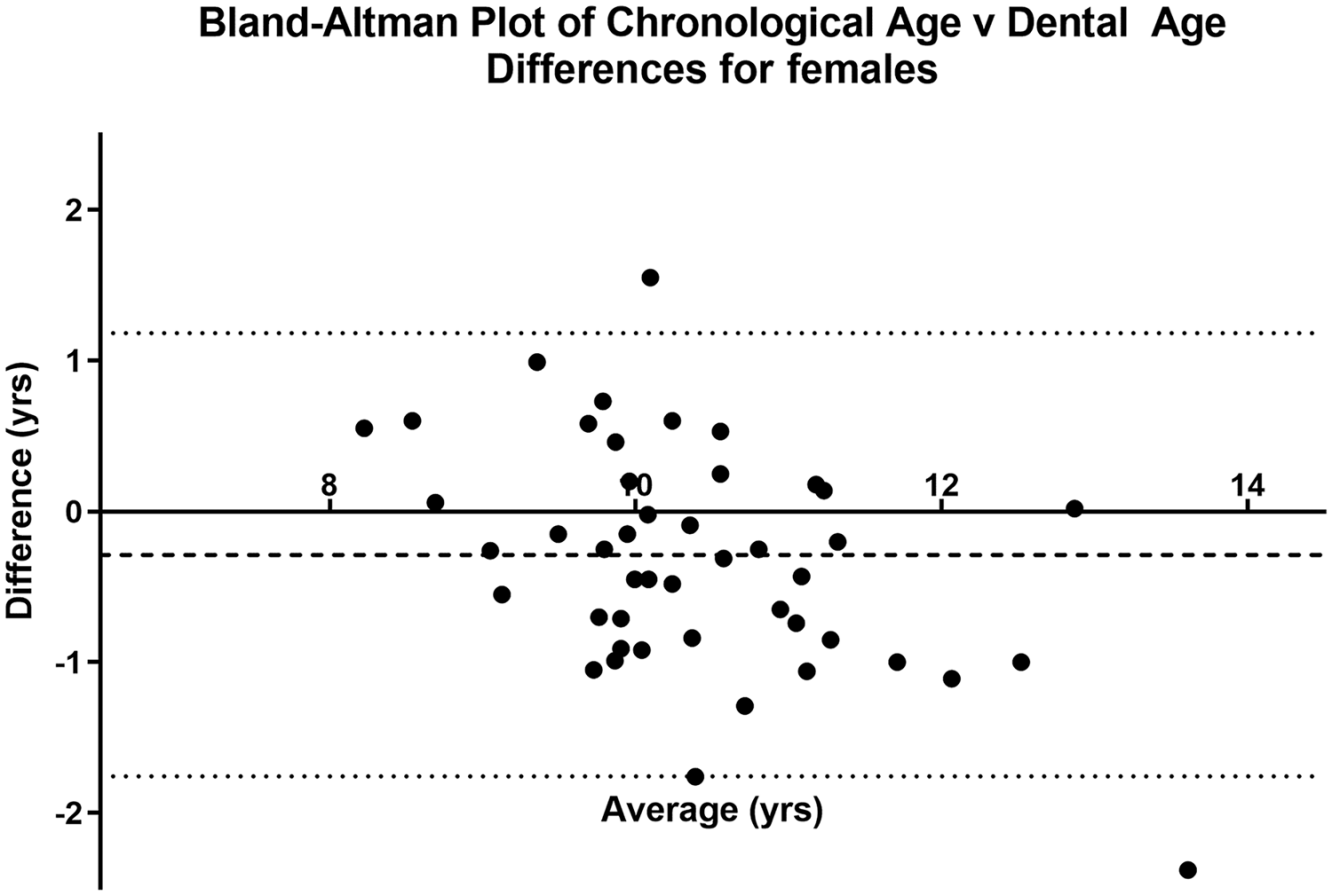
Bland–Altman plot demonstrating that the spread of DA values is evenly distributed around the mean value of the CA (for females)

**Fig. 5:**
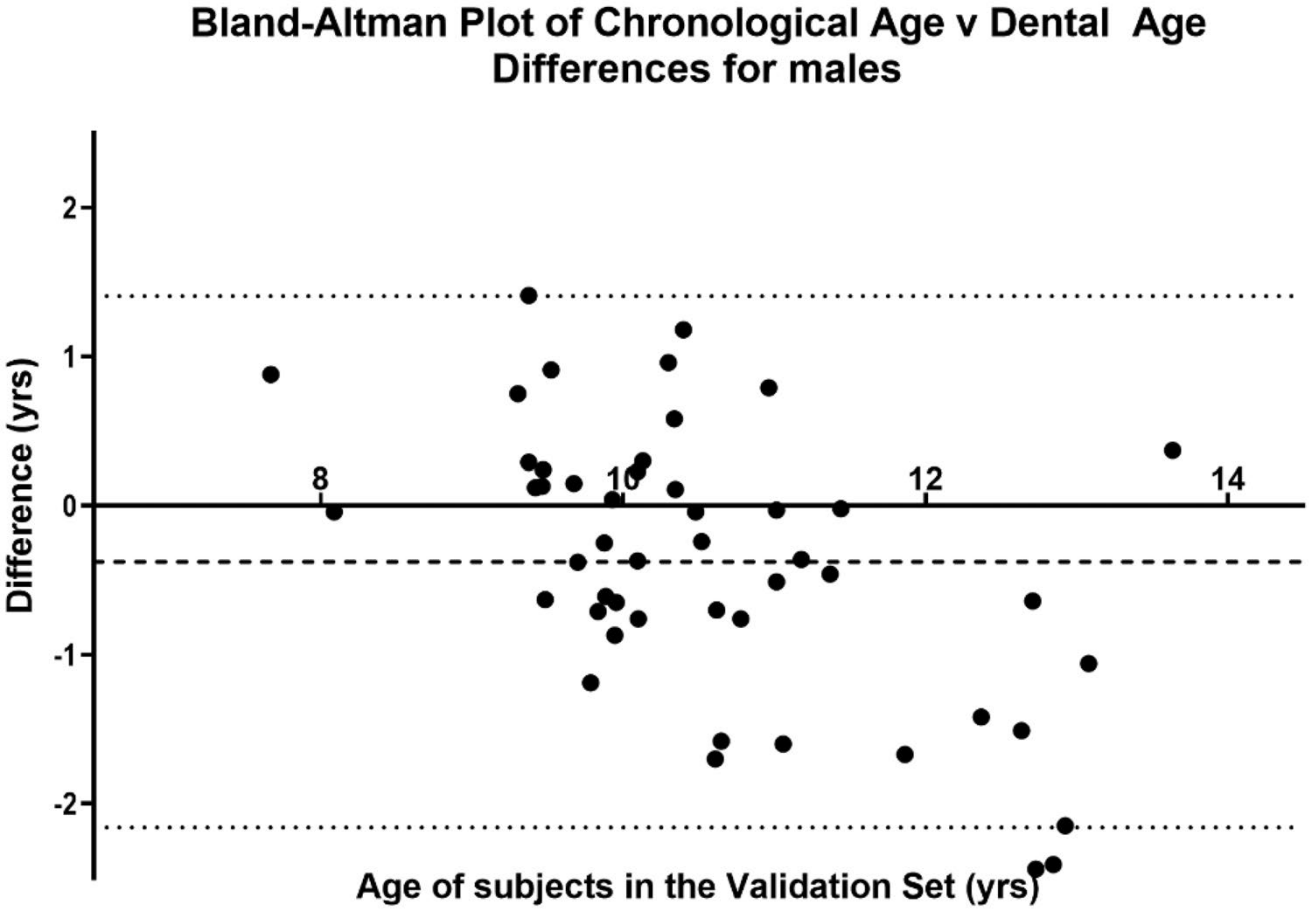
Bland–Altman plot demonstrating that the spread of DA values is evenly distributed around the mean value of the CA (for males)

The distribution of individual TDS for subject 47f, gives the estimated DA with the variation of the individual TDS (Fig. 3). In addition to this formal statistical test, Bland–Altman graphs were used to show the variation of the difference between CA and DA around the line of no difference (Figs. 4 and 5) [7].

## Results

### Sample distribution

The number of female and male subjects by 1 year age bands is shown in Table 2.

**Table 2:** Numbers of subjects contributing radiographs by 1 year age spans A total of 1,597 subjects spread fairly evenly between females and males comprised the DPT from subjects resident in Qatar

**Age span**	**Number of females**	**Number of males**
6.00–6.99	2	4
7.00–7.99	21	19
8.00–8.99	48	47
9.00–9.99	42	59
10.00–10.99	123	86
11.00–11.99	122	110
12.00–12.99	80	92
13.00–13.99	40	99
14.00–14.99	31	47
15.00–15.99	17	58
16.00–16.99	20	32
17.00–17.99	14	54
18.00–18.99	21	15
19.00–19.99	19	11
20.00–20.99	22	8
21.00–21.99	27	10
22.00–22.99	39	11
23.00–23.99	31	18
24.00–24.99	26	16
25.00–25.99	37	19
**Total**	782	815

### Observer agreement

The WOA score for TDS was 0.9203 and the BOA score for TDS was 0.8401 demonstrating an almost perfect agreement [5].

### Reference data set

Radiographs of 1597 Qatari children, adolescents, and young adults age between 5 and 25 years were assessed by the main investigator (NAO) to create the RDS. The data for females and males across the whole range of the age are shown in Table 3 comprising normal distribution summary statistics and percentile distribution summary statistics.

**Table 3:** Qatari RDS for 782 females and 815 males

	All numerical data in years							
**TDS**	**n-tds**	$\overline{{\varvec{x}} }$**-tds**	**sd-tds**	**0%ile (min)**	**25**^**th**^**%ile**	**50**^**th**^**%ile**	**75%ile**	**100%ile (max)**
UR8Af	5	9.67	0.54	8.99	9.42	9.52	10.16	10.28
UR8Am	8	9.50	0.78	8.02	9.13	9.91	9.98	9.98
	*Difference f minus m* = *0.17, 95% CL* = *−0.711 to 1.051, t* = *0.425, p* > *0.679 ns*							
UR8Bf	50	9.90	1.23	7.47	8.97	9.89	10.63	12.51
UR8Bm	37	9.98	0.97	8.12	9.18	9.98	10.54	11.90
	*Difference f minus m* = *−0.08, 95% CI* = *−0.566 to 0.406, t* = *−0.327, p* > *0.744 ns*							
UR8Cf	85	10.89	1.16	8.19	10.16	10.83	11.51	13.54
UR8Cm	106	11.15	1.16	8.52	10.38	11.18	11.89	13.65
	*Difference f minus m* = *−0.26, 95% CI* = *−0.593 to 0.073, t* = *−1.539, p* > *0.125 ns*							
UR8Df	156	12.13	1.45	9.17	11.09	12.12	12.89	18.25
UR8Dm	173	12.65	1.48	8.58	11.60	12.79	13.43	18.12
	*Difference f minus m* = *−0.52, 95% CI* = *−0.838 to −0.201, t* = *−3.212, p* > *0.001 ns*							
UR8Ef	38	13.73	1.14	11.52	12.86	13.85	14.49	16.19
UR8Em	93	14.03	1.43	11.26	13.30	13.81	14.69	18.64
	*Difference f minus m* = *−0.3, 95% CI* = *−0.815 to 0.215, t* = *−1.15, p* > *0.251 ns*							
UR8Ff	24	16.23	1.62	13.71	14.82	16.21	17.34	19.19
R8Fm	39	15.80	1.32	13.32	15.20	15.75	16.98	19.19
	*Difference f minus m* = *0.43, 95% CI* = *−0.32 to 1.18, t* = *1.15, p* > *0.25 ns*							
UR8Gf	33	17.75	2.05	14.81	16.28	17.10	18.92	21.98
UR8Gm	65	16.61	1.22	11.76	15.96	16.75	17.37	18.93
	*Difference f minus m* = *1.14, 95% CI* = *0.48 to 1.79, t* = *3.45, p* > *0.00 ns*							
UL1Ef	1	7.51		7.52	7.52	7.52	7.52	7.52
UL1Em	4	7.94	0.80	7.14	7.25	7.98	8.63	8.65
	*Unable to compute*							
UL1Ff	26	7.86	0.67	6.86	7.36	7.86	8.24	9.42
UL1Fm	33	8.04	0.72	6.32	7.67	8.05	8.53	9.67
	*Difference f minus m* = *−0.18, 95% CL* = *−0.547 to 0.187, t* = *−0.983, p* > *0.33 ns*							
UL1Gf	26	8.63	0.79	7.47	8.20	8.53	8.88	10.38
UL1Gm	39	8.95	0.97	7.21	8.28	8.88	9.80	10.83
	*Difference f minus m* = *−0.32, 95% CI* = *−0.77 to 0.14, t* = *−1.39, p* > *0.166 ns*							
UL2Df								
UL2Dm	2	7.70	0.48	7.35	7.35	7.70	8.04	8.04
	*Unable to compute*							
UL2Ef	9	7.30	0.30	6.86	7.13	7.25	7.52	7.78
UL2Em	18	7.95	0.68	6.32	7.66	8.13	8.53	8.65
	*Difference f minus m* = *−0.65, 95% CI* = *−1.14 to −0.16, t* = *−2.72, p* > *0.012 ns*							
UL2Ff	32	8.25	0.56	7.22	7.93	8.25	8.67	9.42
UL2Fm	47	8.56	0.92	6.87	8.02	8.52	9.18	10.83
	*Difference f minus m* = *−0.31, 95% CI* = *−0.67 to 0.05, t* = *−1.70, p* > *0.092 ns*							
**TDS**	**n- tds**	$\overline{{\varvec{x}} }$**-tds**	**sd-tds**	**0%ile (min)**	**25**^**th**^**%ile**	**50**^**th**^**%ile**	**75%ile**	**100%ile (max)**
UL2Gf	47	9.53	1.01	7.57	8.59	9.42	10.31	11.92
UL2Gm	75	10.01	0.89	7.86	9.32	10.12	10.66	11.92
	*Difference f minus m* = *−0.48, 95% CI* = *−0.83 to −0.13, t* = *−2.75, p* > *0.006 ns*							
UL3Df	2	7.32	0.27	7.13	7.13	7.32	7.52	7.52
UL3Dm	2	7.29	0.09	7.23	7.23	7.29	7.35	7.35
	*Difference f minus m* = *0.03, 95% CL* = *−0.84 to 0.89, t* = *0.15, p* > *0.895 ns*							
UL3Ef	19	7.76	0.59	6.86	7.25	7.64	8.20	8.95
UL3Em	41	8.09	0.71	6.32	7.66	8.11	8.62	9.35
	*Difference f minus m* = *−0.33, 95% CI* = *−0.71 to 0.05, t* = *−1.76, p* > *0.083 ns*							
UL3Ff	94	9.39	1.09	7.36	8.56	9.21	10.26	12.13
UL3Fm	156	9.94	0.96	7.74	9.19	10.05	10.54	11.90
	*Difference f minus m* = *−0.55, 95% CI* = *−0.81 to −0.29, t* = *−4.17, p* > *0 ns*							
UL3Gf	208	10.82	0.81	0.81	8.26	10.22	10.86	13.23
UL3Gm	141	11.51	0.92	8.98	11.01	11.49	12.06	13.99
	*Difference f minus m* = *−0.69, 95% CI* = *−0.87 to −0.51, t* = *−7.34, p* > *0 ns*							
UL4Df	13	7.61	0.62	6.86	7.22	7.40	7.78	8.88
UL4Dm	23	7.86	0.73	6.32	7.22	7.94	8.47	9.32
	*Difference f minus m* = *−0.25, 95% CI* = *−0.74 to 0.24, t* = *−1.04, p* > *0.31 ns*							
UL4Ef	37	8.54	0.83	6.99	8.11	8.39	8.81	10.38
UL4Em	56	8.69	0.79	6.97	8.22	8.61	9.21	10.32
	*Difference f minus m* = *−0.15, 95% CI* = *−0.49 to 0.19, t* = *−0.88, p* > *0.38 ns*							
UL4Ff	76	9.57	0.85	7.89	8.93	9.61	10.25	11.29
UL4Fm	78	9.89	0.73	7.86	9.23	9.98	10.29	11.69
	*Difference f minus m* = *−0.32, 95% CI* = *−0.57 to −0.067, t* = *−2.51, p* > *0.013 ns*							
UL4Gf	109	10.77	0.81	8.86	10.22	10.64	11.29	13.36
UL4Gm	93	10.98	0.80	8.84	10.45	10.96	11.50	13.35
	*Difference f minus m* = *−0.21, 95% CI* = *−0.43 to 0.01, t* = *−1.85, p* > *0.067 ns*							
UL5Df	13	7.68	0.72	6.86	7.15	7.37	8.24	8.88
UL5Dm	21	7.84	0.76	6.32	7.23	7.86	8.47	9.33
	*Difference f minus m* = *−0.16, 95% CI* = *−0.69 to 0.38, t* = *−0.61, p* > *0.55 ns*							
UL5Ef	54	8.83	0.95	7.36	8.13	8.58	9.59	10.49
UL5Em	78	9.06	0.99	6.97	8.28	8.98	9.97	11.67
	*Difference f minus m* = *−0.23, 95% CI* = *−0.57 to 0.11, t* = *−1.33, p* > *0.18 ns*							
UL5Ff	118	10.26	0.98	7.89	9.64	10.31	11.08	12.68
UL5Fm	120	10.39	0.89	8.16	9.82	10.40	11.01	12.09
	*Difference f minus m* = *−0.13, 95% CL* = *−0.37 to0.11, t* = *−1.07, p* > *0.29 ns*							
UL5Gf	151	11.15	0.94	8.86	10.38	11.09	11.92	13.36
UL5Gm	100	11.59	0.95	8.84	11.04	11.61	12.31	13.81
	*Difference f minus m* = *−0.44, 95% CI* = *−0.68 to −0.20, t* = *−3.62, p* > *0.00 ns*							
**TDS**	**n- tds**	$\overline{{\varvec{x}} }$**-tds**	**sd-tds**	**0%ile (min)**	**25**^**th**^**%ile**	**50**^**th**^**%ile**	**75%ile**	**100%ile (max)**
UL6Ef								
UL6Em	1	6.32		6.32	6.32	6.32	6.32	6.32
	*Unable to compute*							
UL6Ff	2	7.39	0.36	7.13	7.13	7.39	7.64	7.64
UL6Fm	1	8.05	0.76	8.05	8.05	8.05	8.05	8.05
	*Unable to compute*							
UL6Gf	25	7.93	0.74	6.99	7.40	7.78	8.30	10.38
UL6Gm	33	8.11	0.85	7.03	7.41	8.11	8.56	11.67
	*Difference f minus m* = *−0.18, 95% CI* = *−0.61 to 0.25, t* = *0.84, p* > *0.40 ns*							
UL7Df	24	7.91	0.76	6.86	7.31	7.71	8.27	9.59
UL7Dm	42	8.22	0.97	6.32	7.66	8.08	8.72	11.67
	*Difference f minus m* = *−0.31, 95% CI* = *−0.77 to 0.15, t* = *−1.35, p* > *0.18 ns*							
UL7Ef	108	9.56	1.16	7.36	8.64	9.56	10.25	14.51
UL7Em	129	9.81	1.08	7.03	9.06	9.95	10.38	13.35
	*Difference f minus m* = *−0.25, 95% CI* = *−0.54 to 0.04, t* = *−1.72, p* > *0.09 ns*							
UL7Ff	100	10.74	0.96	8.77	10.25	10.61	11.27	13.36
UL7Fm	73	10.91	0.99	8.58	10.34	10.96	11.62	12.77
	*Difference f minus m* = *−0.17, 95% CI* = *−0.47 to 0.13, t* = *−1.14, p* > *0.26 ns*							
UL7Gf	167	11.49	0.98	9.61	10.80	11.49	12.13	16.95
UL7Gm	173	12.20	1.12	9.06	11.39	12.11	13.03	15.57
	*Difference f minus m* = *−0.71, 95% CI* = *−0.93 to −0.49, t* = *−6.21, p* > *0.0 ns*							
UL8Af	5	9.35	0.57	8.65	8.99	9.42	9.52	10.16
UL8Am	9	9.78	1.02	8.02	9.40	9.91	10.19	11.17
	*Difference f minus m* = *−0.43, 95% CL* = *−1.52 to0.66, t* = *−0.86, p* > *0.41 ns*							
UL8Bf	47	9.83	1.14	7.47	8.97	9.92	10.62	12.35
UL8Bm	36	10.13	1.19	8.12	9.20	9.98	10.88	13.19
	*Difference f minus m* = *−0.3, 95% CI* = *−0.81 to 0.21, t* = *−1.17, p* > *0.25 ns*							
UL8Cf	95	10.93	1.16	8.19	10.16	10.89	11.54	13.65
UL8Cm	106	11.06	1.10	8.67	10.29	11.03	11.88	14.04
	*Difference f minus m* = *−0.13, 95% CI* = *−0.44 to 0.18, t* = *−0.82, p* > *0.42 ns*							
UL8Df	149	12.13	1.37	9.17	11.13	12.14	12.84	17.70
UL8Dm	178	12.69	1.62	8.12	11.60	12.79	13.49	18.97
	*Difference f minus m* = *−0.56, 95% CI* = *−0.89 to −0.23, t* = *−3.34, p* > *0.00 ns*							
UL8Ef	41	13.89	1.39	11.52	12.93	13.86	14.79	19.19
UL8Em	94	14.00	1.39	11.26	13.30	13.80	14.68	18.64
	*Difference f minus m* = *−0.11, 95% CI* = *−0.62 to 0.40, t* = *−0.42, p* > *0.67 ns*							
UL8Ff	19	16.03	1.57	13.65	14.59	16.00	17.24	19.69
UL8Fm	36	15.76	1.13	13.32	15.30	15.75	16.81	17.45
	*Difference f minus m* = *0.27, 95% CI* = *−0.47 to 1.00, t* = *0.73, p* > *0.47 ns*							
**TDS**	**n- tds**	$\overline{{\varvec{x}} }$**-tds**	**sd-tds**	**0%ile (min)**	**25**^**th**^**%ile**	**50**^**th**^**%ile**	**75%ile**	**100%ile (max)**
UL8Gf	32	17.51	2.00	14.81	16.19	16.88	18.57	21.97
UL8Gm	64	16.68	1.06	14.44	15.96	16.70	17.36	18.93
	*Difference f minus m* = *0.83, 95% CI* = *0.21 to 1.45, t* = *2.67, p* > *0.01 ns*							
LR8Af	35	9.89	1.25	7.47	8.94	10.08	11.29	11.92
LR8Am	26	9.69	1.15	7.77	8.68	10.07	10.46	11.44
	*Difference f minus m* = *0.2, 95% CI* = *−0.43 to 0.83, t* = *0.64, p* > *0.53 ns*							
LR8Bf	46	9.97	1.22	7.93	8.95	10.11	10.50	13.08
LR8Bm	32	10.01	1.07	8.12	9.28	9.98	10.76	12.69
	*Difference f minus m* = *−0.04, 95% CI* = *−0.57 to 0.49, t* = *−0.15, p* > *0.88 ns*							
LR8Cf	95	10.90	1.14	8.19	10.16	10.62	11.64	13.65
LR8Cm	107	10.92	1.30	8.52	10.05	10.67	11.75	
	*Difference f minus m* = *−0.02, 95% CI* = *−0.36 to 0.32, t* = *−0.12, p* > *0.91 ns*							
LR8Df	176	12.11	1.39	9.17	11.16	11.95	12.88	18.25
LR8Dm	208	12.68	1.43	8.58	11.61	12.77	13.51	18.53
	*Difference f minus m* = *−0.57, 95% CI* = *−0.85 to – 0.29, t* = *-3.94, p* > *0.00 ns*							
LR8Ef	37	14.21	1.33	11.97	13.30	14.13	14.85	18.39
LR8Em	78	14.27	1.37	11.40	13.45	13.84	15.20	18.64
	*Difference f minus m* = *−0.06, 95% CI* = *−0.59 to 0.48, t* = *−0.22, p* > *0.83 ns*							
LR8Ff	20	16.07	1.78	13.65	14.70	15.58	17.21	20.48
LR8Fm	37	15.49	1.24	12.94	14.48	15.62	16.60	17.59
	*Difference f minus m* = *0.58, 95% CL* = *−0.23 to1.39, t* = *1.44, p* > *0.155 ns*							
LR8Gf	37	17.67	1.97	14.36	16.25	17.10	19.26	21.37
LR8Gm	71	16.82	1.23	14.36	15.57	17.07	17.41	20.81
	*Difference f minus m* = *0.58, 95% CL* = *−0.24 to 1.46, t* = *2.75, p* > *0.01 ns*							
LL1Ff	5	7.35	0.51	6.86	6.99	7.25	7.52	8.15
LL1Fm	6	7.44	0.67	6.87	6.97	7.18	7.77	8.65
	*Difference f minus m* = *−0.09, 95% CI* = *−0.92 to 0.74, t* = *−0.25, p* > *0.81 ns*							
LL1Gf	13	7.86	0.63	7.13	7.40	7.78	8.24	9.42
LL1Gm	25	7.89	0.62	6.32	7.35	8.02	8.29	8.71
	*Difference f minus m* = *−0.03, 95% CI* = *−0.46 to 0.40, t* = *−0.14, p* > *0.89 ns*							
LL2Ff	13	7.68	0.71	6.86	7.15	7.44	8.13	9.42
LL2Fm	28	7.93	0.74	6.32	7.32	7.98	8.41	9.33
	*Difference f minus m* = *−0.25, 95% CI* = *−0.74 to 0.25, t* = *−1.02, p* > *0.31 ns*							
LL2Gf	33	8.48	0.81	7.36	7.93	8.38	8.81	10.38
LL2Gm	28	8.70	0.84	7.03	8.14	8.67	9.31	10.28
	*Difference f minus m* = *−0.22, 95% CI* = *−0.64 to 0.20, t* = *−1.04, p* > *0.30 ns*							
LL3Df								
LL3Dm	1	8.05		8.05	8.05	8.05	8.05	8.05
	*Unable to compute*							
LL3Ef	14	7.63	0.53	6.86	7.15	7.58	8.15	8.38
LL3Em	33	7.96	0.70	6.32	7.35	7.95	8.47	9.33
	*Difference f minus m* = *0.33, 95% CL* = *−0.75 to 0.09, t* = *−1.58, p* > *0.12 ns*							
**TDS**	**n- tds**	$\overline{{\varvec{x}} }$**-tds**	**sd-tds**	**0%ile (min)**	**25**^**th**^**%ile**	**50**^**th**^**%ile**	**75%ile**	**100%ile (max)**
LL3Ff	87	9.08	1.01	7.22	8.29	8.94	9.86	11.42
LL3Fm	136	9.60	0.94	6.87	8.98	9.84	10.22	11.90
	*Difference f minus m* = *−0.52, 95% CL* = *−0.78 to -0.26, t* = *−3.91, p* > *0.00 ns*							
LL3Gf	163	11.61	0.81	8.63	10.08	10.48	11.24	12.92
LL3Gm	123	11.21	0.84	8.84	10.64	11.24	11.64	13.99
	*Difference f minus m* = *5.4, 95% CL* = *5.21 to 5.59, t* = *54.93, p* > *0.00 ns*							
LL4Df	1	7.22		7.22	7.22	7.22	7.22	7.22
LL4Dm	3	7.40	0.94	6.32	6.32	7.86	8.02	8.02
	*unable to compute*							
LL4Ef	43	8.14	0.74	6.86	7.47	8.21	8.53	10.26
LL4Em	57	8.32	0.78	6.87	7.86	8.28	8.72	10.07
	*Difference f minus m* = *−0.18, 95% CL* = *−0.49 to 0.13, t* = *−1.17, p* > *0.25 ns*							
LL4Ff	101	9.77	0.96	7.89	8.99	9.85	10.31	12.68
LL4Fm	122	9.98	0.92	7.31	9.26	10.00	10.66	11.90
	*Difference f minus m* = *−0.21, 95% CL* = *−0.46 to 0.04, t* = *−1.66, p* > *0.09 ns*							
LL4Gf	167	10.94	0.75	9.32	10.38	10.93	11.42	13.36
	134	11.34	0.93	9.06	10.64	11.34	11.90	13.99
	*Difference f minus m* = *−0.4, 95% CL* = *−0.59 to −0.21, t* = *−4.13, p* > *0.00 ns*							
LL5Df	10	7.89	0.90	6.86	7.13	7.74	8.68	9.40
LL5Dm	20	7.82	0.67	6.32	7.25	7.94	8.28	9.08
	*Difference f minus m* = *0.07, 95% CL* = *−0.53 to 0.67, t* = *0.24, p* > *0.81 ns*							
LL5Ef	55	8.56	0.86	7.15	7.95	8.41	8.95	10.36
LL5Em	79	9.12	1.09	6.87	8.26	8.98	10.07	11.44
	*Difference f minus m* = *−0.56, 95% CL* = *−0.91 to −0.21, t* = = *3.18, p* > *0.00 ns*							
LL5Ff	172	10.42	0.95	8.03	9.95	10.40	11.08	13.08
LL5Fm	169	10.65	1.05	8.52	9.98	10.64	11.32	14.05
	*Difference f minus m* = *−0.23, 95% CL* = *−0.44 to −0.02, t* = *−2.12, p* > *0.03 ns*							
LL5Gf	134	11.44	0.88	9.61	10.83	11.39	11.94	13.82
LL5Gm	104	12.06	1.04	9.18	11.49	12.09	12.74	13.99
	*Difference f minus m* = *−0.62, 95% CL* = *−0.87 to −0.37, t* = *−4.98, p* > *0.0 ns*							
LL6Ff	1	7.13		7.13	7.13	7.13	7.13	7.13
LL6Fm	2	8.84	0.77	8.29	8.29	8.84	9.39	9.39
	*Unable to compute*							
LL6Gf	35	7.99	0.68	6.86	7.47	8.11	8.30	10.38
LL6Gm	52	8.34	1.00	6.32	7.81	8.25	8.71	11.89
	*Difference f minus m* = *−0.35, 95% CL* = *−0.74 to 0.04, t* = *−1.81, p* > *0.07 ns*							
LL7Cf								
LL7Cm	1	11.78		11.78	11.78	11.78	11.78	11.78
	*Unable to compute*							
**TDS**	**n- tds**	$\overline{{\varvec{x}} }$**-tds**	**sd-tds**	**0%ile (min)**	**25**^**th**^**%ile**	**50**^**th**^**%ile**	**75%ile**	**100%ile (max)**
LL7Df	26	7.86	0.65	6.86	7.37	7.71	8.29	9.40
LL7Dm	42	8.26	1.26	6.32	7.35	8.04	8.65	11.86
	*Difference f minus m* = *−0.4, 95% CL* = *−0.93 to 0.13, t* = *−1.49, p* > *0.14 ns*							
LL7Ef	77	9.46	1.09	7.36	8.56	9.42	10.26	12.13
LL7Em	89	9.56	0.94	7.34	8.76	9.66	10.18	11.65
	*Difference f minus m* = *−0.1, 95% CL* = *−0.41 to 0.21, t* = *−0.63, p* > *0.53 ns*							
LL7Ff	102	10.29	0.92	8.19	9.72	10.26	10.78	13.08
LL7Fm	94	10.52	0.96	8.58	9.98	10.40	11.27	12.76
	*Difference f minus m* = *−0.23, 95% CL* = *−0.49 to 0.03, t* = *−4.98, p* > *0.0 ns*							
LL7Gf	222	11.65	0.98	9.98	11.01	11.51	12.21	14.66
LL7Gm	226	12.27	1.16	9.18	11.44	12.19	13.14	15.86
	*Difference f minus m* = *2.1, 95% CL* = *1.89 to 2.3, t* = *20.47, p* > *0.0 ns*							
LL8Af	39	10.11	1.18	7.52	9.42	10.09	11.29	12.59
LL8Am	28	9.55	1.18	7.67	8.61	9.39	10.34	11.44
	*Difference f minus m* = *0.56, 95% CL* = *−0.02 to 1.14, t* = *1.92, p* > *0.06 ns*							
LL8Bf	43	9.67	0.94	7.93	8.88	9.92	10.48	12.04
LL8Bm	33	10.39	1.31	8.12	9.57	10.00	11.18	13.43
	*Difference f minus m* = *−0.72, 95% CL* = *−1.23 to −0.21, t* = *−2.79, p* > *0.01 ns*							
LL8Cf	83	10.76	1.05	8.19	10.10	10.61	11.48	13.36
LL8Cm	111	10.98	1.35	8.52	10.06	10.67	11.90	15.02
	*Difference f minus m* = −*0.22, 95% CL* = *−0.57 to 0.13, t* = *−1.23, p* > *0.22 ns*							
LL8Df	172	12.01	1.18	9.17	11.56	11.90	12.79	15.33
LL8Dm	207	12.68	1.48	8.58	11.60	12.76	13.52	17.13
	*Difference f minus m* = *−0.67, 95% CL* = *−0.94 to – 0.39, t* = *−4.80, p* > *0.0 ns*							
LL8Ef	37	14.39	1.56	11.97	13.51	14.13	14.94	18.39
LL8Em	80	14.26	1.35	11.40	13.45	13.84	15.21	18.64
	*Difference f minus m* = *0.13, 95% CL* = *−0.43 to 0.69, t* = *0.46, p* > *0.65 ns*							
LL8Ff	26	15.90	1.72	12.93	14.81	15.50	16.95	19.52
LL8Fm	35	15.47	1.13	13.32	14.48	15.47	16.33	17.59
	*Difference f minus m* = *0.43, 95% CL* = −*0.30 to1.161, t* = *1.178, p* > *0.244 ns*							
LL8Gf	39	18.00	2.08	14.36	16.25	17.44	20.14	21.37
LL8Gm	68	16.84	1.22	14.36	15.96	17.10	17.43	20.81
	*Difference f minus m* = *1.16, 95% CL* = *0.53 to1.79, t* = *3.64, p* > *0.00 ns*							
**TDS**	**n- tds**	$\overline{{\varvec{x}} }$**-tds**	**sd-tds**	**0%ile (min)**	**25**^**th**^**%ile**	**50**^**th**^**%ile**	**75%ile**	**100%ile (max)**

### Worked example

The detailed procedure for the computer-assisted procedure to estimate individual DA is detailed in a paper published in 2018 [6].

### Validation study

The data extracted for each subject are illustrated in the worked example (Table 4). The VS of 100 subjects comprise 50 females and 50 males age between 5 and 25 years with known date of birth and date of radiographic exposure. The results from the calculations for the whole of the VS are shown separately for females (Fig. 4) and males (Fig. 5).

**Table 4:** Worked example. Data extracted for subject 47. The Demirjian TDS are indicated as B, E, F, G, H. The data for these stages are extracted from the RDS (Table 3). *AaA*, age at assessment

**Tooth (anatomical description)**	**FDI notation**	**Demirjian TDS**	**AaA** $\overline{{\varvec{x}} }$	**AaA** **sd**	**BrDJ notation**
Upper left central incisor	21	H	-	-	UL1
Upper left lateral incisor	22	G	9.53	1.01	UL2
Upper left canine	23	F	9.39	1.09	UL3
Upper left first premolar	24	F	9.57	0.85	UL4
Upper left second premolar	25	E	8.83	0.95	UL5
Upper left first permanent molar	26	H	-	-	UL6
Upper left second permanent molar	27	E	9.56	1.16	UL7
Upper left third molar	28	-	-	-	UL8
Lower left third molar	38	B	9.67	0.94	LL8
Lower left second permanent molar	37	F	10.29	0.92	LL7
Lower left first permanent molar	36	H	-	-	LL6
Lower left second premolar	35	F	10.42	0.95	LL5
Lower left first premolar	34	F	9.77	0.96	LL4
Lower left canine	33	F	9.08	1.01	LL3
Lower left lateral incisor	32	H	-	-	LL2
Lower left central incisor	31	H	-	-	LL1

Dental age = 9.61 years; sd = 0.98; chronological age = 10.41 years

The mean CA for females was 10.8 years, sd 1.74 years, the lower confidence limit was 10.31 years, and the upper confidence limit was 11.29 years.

The mean difference between CA and estimated DA is 0.40 years or an overestimate of 4.84 months (Table 5).

**Table 5:** The validation sample comparison of CA and DA for females

Variable [females]	Observation	$\overline{\mathrm{x} }$(years)	sd	Limits of 95% conf. interval	
CAf	50	10.8	1.74	10.31	11.29
DAf	50	10.4	1.33	10.03	10.78
Difference	50	0.40	0.93	0.14	0.67
*t*			3.06		
Degrees of freedom			49		
*p* value			0.0036		
Difference in months			4.84		

The mean CA for males was 10.89 years, sd 1.82 years, the lower confidence limit was 10.37 years, and the upper confidence limit was 11.41 years.

The mean difference between CA and DA for males is an overestimate of 0.38 years or 4.55 months (Table 6).

**Table 6:** The validation sample comparison of CA and DA for males

Variable [males]	Observation	$\overline{\mathrm{x} }$(years)	sd	Limits of 95% conf. interval	
CAm	50	10.89	1.82	10.37	11.41
DAm	50	10.52	1.42	10.11	10.92
Difference	50	0.38	0.91	0.12	0.64
*t*			2.49		
Degrees of freedom			49		
*p* value			0.0050		
Difference in months			4.55		

### Bland–Altman graphs

These graphs [7] explore the agreement between CA and DA for both female and male subjects. The graphs (Figs. 4 and 5) demonstrate the difference between the CA and DA within the female and male VS groups. The horizontal dotted line is located at the mean difference between the CA and DA whilst the continuous line represents the “no difference” value between CA and DA. In both the female and male groups, the mean difference was statistically significant.

## Discussion

The aim of DAE is to estimate the chronological age of an individual with unknown date of birth. It may be used for clinical, legal, civil, criminal, and forensic purposes. An important consideration is the error of the method. The preferred term is the uncertainty associated with the estimate. This is easily and reliably achieved using Draft Quicksheets [6].

In the present study, only the tooth development stages, which comprise the first part of the Demirjian, Goldstein, and Tanner method [2], were employed. This is because the mathematical procedures leading to the maturity score *cannot* be unravelled [8]. In the SAM procedure, all the developing teeth on the left side including third molars were used. This has increased the number of assessed teeth and therefore likely to lead to an improvement in the accuracy or reliability of the estimated age as it uses the maximum information available. This is born out to a considerable extent by the relatively small CA *minus* DA differences shown in the results of the validation study. In addition, the inclusion of third molars up to and including stage G makes the method suitable for older subjects aged 16 years or more. Stage H is not used as it is unbounded in its upper border for each tooth. This will be the subject of a further communication because the age of apex closure cannot be identified without censoring [8]. Also, the mean value for stage H cannot be used in simple mathematical procedures such as averaging the AaA of TDSs present in a subject. Therefore stage H is excluded from any calculations in SAM.

Each ethnicity or ancestral group has its own characteristics including with DAE. Many studies investigate the validity of using the French-Canadian standards on other populations [9–11]. However, it was found that overestimation or underestimation of age was present in many studies. From those findings developed the concept of establishing ethnic specific RDSs which is important for each specific Identifiable Human Group or Ancestral Group.

In this study, a RDS with the mean AaA of different TDS for the Qatari population was developed. The mean age at attainment is the term often used in DAE research reports. This is logically inconsistent as the age at which a stage has been attained *cannot* be ascertained from a single “snapshot” radiograph of developing teeth. Age at assessment (AaA) is the logical and better understood term.

The data collection was limited to Qatari subjects and partitioned by sex. The large number of DPTs results in a sufficiently large number in each 1 year age span and consequently for the individual TDS to ensure a narrow range of the CI of the differences between females and males. In practice, over 73% of the AaA values for the individual TDS were earlier in females. This emphasizes the importance of sex-specific data in addition to the need for ethnic specific data.

This work is part of a large dental age estimation project looking at different ancestral or identifiable human groups [6, 12, 13]. The main purpose of the study was to establish a RDS for the Qatari population. This was achieved using the 8 stage system of TDSs. A strong justification for this is that it has been shown to return the highest WOA and BOA using Cohen’s kappa. The scheme is practical, reliable, and easy to use as has been shown by the German research team [14]. Other publications using SAM have shown consistent accuracy within 6 months of the CA [13, 15].

The second part of the Demirjian, Goldstein, and Tanner method [2] has not been used as it has been suggested that age estimates based on the French-Canadian population are not applicable to other populations [2]. The method used here has been named as the simple average method (SAM) as it uses all the tooth morphology types without any idiosyncratic weighting, and on logical grounds, it is intuitively more informative to use as much information by using all tooth morphology types available [6].

A simple assessment to emphasize the need for ancestral specific data is to compare two TDS at random: Kuwait LL7Gf cf Qatar LL7Gf. These two stages from different Middle Eastern populations provide data as follows – Kuwait LL7Gf n-tds = 95, x-tds = 13.56, sd-tds = 1.55; Qatar LL7Gf n-tds = 222, x-tds = 11.65, sd-tds = 0.98. The difference of 1.91 years is highly statistically different. Whether this applies to the other TDSs in these RDSs is a matter for future investigation and report. This raises the important question of the suitability of using apparently similar genetic backgrounds for DAE.

The robust nature of the present study has been supported by the following:

1. A single identifiable human group – Qatari subjects – has been used to create the reference data set.
2. Training and careful management of the WOA and BOA supports the use of the 8 stage TDS system.
3. A validation set was used to test the accuracy of the RDS when used to estimate the dental age of Qatari children, adolescents, and young adults.

## Conclusion

A reference data set for the mean age at assessment of the different TDS for the Qatari population has been established. The average difference between CA and DA for female and male subjects was 4.8 months and 4.5 months, respectively.

## Key points

1. A large reference data set has been established for Qatari subjects.
2. There is a clear difference between Age at Assessment for females and males as regards Tooth Development Stages.
3. The Simple Average Method provides age estimation within 5 months of the Chronological Age.

## Data Availability

All primary data is available from the corresponding author.

## References

[1] United Nations Convention on the Rights of the Child. 1989. www.unicef.org/child-rights-convention/convention-text-children’s-version

[2] Demirjian A, Goldstein H, Tanner JM (1973). A new system of dental age assessment. Hum Biol.

[3] Cameriere R, Ferrante L, Liversidge HM, Prieto JL, Brkic H (2008). Accuracy of age estimation in children using radiographs of developing teeth. Forensic Sci Int.

[4] Cohen J (1960). A Coefficient of agreement for nominal scales. Educ Psychol Meas.

[5] Landis JR, Koch GG (1977). The measurement of observer agreement for categorical data. Biometrics.

[6] Draft D, Lucas VS, McDonald F, Andiappan M, Roberts G (2019). Expressing uncertainty in dental age estimation: a comparison between two methods of calculating the ‘average’ standard deviation. J Forensic Sci.

[7] Bland JM, Altman DG. Statistical methods for assessing agreement between two methods of clinical measurement. Lancet. 1986;307–10. 10.1016/S0140-6736(86)90837-8. 2868172

[8] Roberts G, Lucas VS (2012). Is the Demirjian, Goldstein and Tanner method of dental age assessment obsolete? A critical review and re-assessment. Insights Anthropol.

[9] Nykanen R, Espeland L, Kvaal SI, Krogstad O (1998). Validity of the Demirjian method for dental age estimation when applied to Norwegian children. Acta Odontol Scand.

[10] Koshy S, Tandon S (1998). Dental age assessment: the applicability of Demirjian’s method in South Indian children. Forensic Sci Int.

[11] Ambarkova M, Galic I, Vodanovic M, Biocina-Lukenda D, Brkic H (2014). Dental age estimation using Demirjian and Willems methods: cross sectional study on children from the former Yugoslavia Republic of Macedonia. Forensic Sci Int.

[12] Moze K, Roberts GJ (2012). Dental age assessment (DAA) of Afro-Trinidadian children and adolescents: development of a reference data set and comparison with Caucasians resident in London. UK J Leg and Forens Med.

[13] Karimi A, Qudeimat MA, Lucas VS, Roberts G (2021). Dental age estimation: development and validation of a reference data set for Kuwaiti children. Arch Oral Biol..

[14] Olze A, Bilang D, Schmidt S, Wernecke KD, Geserick G, Schmeling A (2005). Validation of common classification systems for assessing the mineralization of third molars. Int J Leg Med.

[15] Mitchel JC, Lucas VS, Roberts GJ (2009). Dental age assessment: reference data for children at the 16 year threshold. Forens Sci Int.

